# Alterations of the murine gut microbiome in allergic airway disease are independent of surfactant protein D

**DOI:** 10.1016/j.heliyon.2017.e00262

**Published:** 2017-03-16

**Authors:** Kenneth K. Barfod, Michael Roggenbuck, Suzan Al-Shuweli, Dalia Fakih, Søren J. Sørensen, Grith L. Sørensen

**Affiliations:** a National Research Centre for the Working Environment, Lersø Parkallé 105, 2100 Copenhagen O, Denmark; b University of Copenhagen, Department of Biology, Microbiology, Universitetsparken 15, 2100 Copenhagen O, Denmark; c Department of Cancer and Inflammation Research, Institute of Molecular Medicine, University of Southern Denmark, Odense, Denmark; d Laboratory of Immunology, Faculty of public health, Lebanese University, Fanar, Lebanon

**Keywords:** Microbiology, Immunology

## Abstract

**Background:**

SP-D is an important host defense lectin in innate immunity and SP-D deficient mice show several abnormal immune effects and are susceptible to allergen-induced airway disease. At the same time, host microbiome interactions play an important role in the development of allergic airway disease, and alterations to gut microbiota have been linked to airway disease through the gut-lung axis. Currently, it is unknown if the genotype (*Sftpd-/- or Sftpd+/+*) of the standard SP-D mouse model can affect the host microbiota to such an degree that it would overcome the cohousing effect on microbiota and interfere with the interpretation of immunological data from the model. Generally, little is known about the effect of the SP-D protein in itself and in combination with airway disease on the microbiota. In this study, we tested the hypothesis that microbiome composition would change with the lack of SP-D protein and presence of allergic airway disease in the widely used SP-D-deficient mouse model.

**Results:**

We describe here for the first time the lung and gut microbiota of the SP-D mouse model with OVA induced allergic airway disease. After the challenge animals were killed and fecal samples were taken from the caecum and lungs were subjected to bronchoalveolar lavage for comparison of gut and lung microbiota by Illumina 16S rRNA gene sequencing. A significant community shift was observed in gut microbiota after challenge with OVA. However, the microbial communities were not significantly different between SP-D deficient and wild type mice from the same cages in either naïve or OVA treated animals. Wild type animals did however show the largest variation between mice.

**Conclusions:**

Our results show that the composition of the microbiota is not influenced by the SP-D deficient genotype under naïve or OVA induced airway disease. However, OVA sensitization and pulmonary challenge did alter the gut microbiota, supporting a bidirectional lung-gut crosstalk. Future mechanistic investigations of the influence of induced allergic airway disease on gut microbiota are warranted.

## 1 Background

Surfactant protein-D (SP-D) belongs to the collectin family of host defense proteins. SP-D is produced by alveolar type II cells in the lung, but is further widely distributed on mucosal surfaces of the body, including the gastrointestinal epithelium [1, 2]. SP-D functions are mainly studied in the lungs. The protein is involved in pulmonary immunity due to lysis, opsonization, neutralization, agglutination, complement activation, enhanced phagocytosis of diverse microbes [3, 4, 5, 6, 7, 8, 9, 10, 11, 12, 13] and the SP-D deficient (*Sftpd-/-*) lung is characterized with inflammatory and structural lung changes resulting in altered lung mechanics [14, 15, 16]. Moreover, airway inflammation and allergic asthma is associated with increases in SP-D levels in bronchoalveolar lavage fluids, tissue, and blood in animal models as well as in human patients, linking SP-D to disease responses [17]. Likewise, induced allergic airway disease in *Sftpd-/-* mice moderately increases the allergic phenotype [18, 19, 20, 21]. In contrast, it is largely unexplored which functions SP-D may have in the intestine, although epithelial uptake of pathogenic bacteria [22] and a disease-modifying role in inflammatory bowel disease is suggested [23, 24].

Novel culture independent techniques for microbial identification have in short of a decade changed the way we view the importance of the microbes that inhabits our bodies. Unique host microbiomes have been associated to priming of the immune system and the development of inflammatory diseases such as asthma, chronic obstructive pulmonary disease (COPD), Crohńs disease, diabetes or obesity [25, 26, 27, 28]. The perinatal priming and development of the microbiome and the putative gut-lung axis have been strongly associated with the development of allergy and asthma [29, 30, 31]. Several animal models have been used to shed light on the mechanisms, although most studies show no evidence of a direct causal effect [32, 33, 34]. Some studies have used fecal transplants to germ-free mice or cross-fostering models in order to show that the pathogenic phenotypes can be ascribed to the gut microbiome [35, 36, 37, 38]. Recently it has been shown that also the lungs harbor complex communities of bacteria in healthy as well as diseased states, which might contribute to pathogenesis [39, 40, 41, 42, 43]. Although, there appear to be a link between inflammatory diseases in the respiratory and intestinal systems, there have been surprisingly few experimental studies investigating a possible cross talk [44, 45]. The most recent major study demonstrate that parasite induced changes in intestinal microbiota leading to changes in the synthesis of anti-inflammatory short chain fatty acids (SCFAs), which have the propensity to dampen development of allergic asthma [46]. On the other hand, allergic asthma may influence the composition of intestinal microbiota as previously demonstrated [40]. Recently we have shown that induction of OVA-induced allergic airway disease itself had a profound effect on the lung microbiome in a vitamin D deficient mouse (BALB/cJ) model [47].

The role of SP-D in regulation of the gut and lung microbiome composition in the setting of pulmonary allergy has not previously been investigated. In this study, we investigate for the first time microbiota of *Sftpd−/−* and *Sftpd+/+* mice, in ovalbumin (OVA) induced airway allergy using Illumina 16S rRNA gene sequencing.

## 2 Results

We have used NGS 16S rRNA gene sequencing to describe lung and gut microbiota of the SP-D mouse model under OVA induced allergic airway disease.

### 2.1 Sequence quality and overall microbial communities

**Fig. 1:**
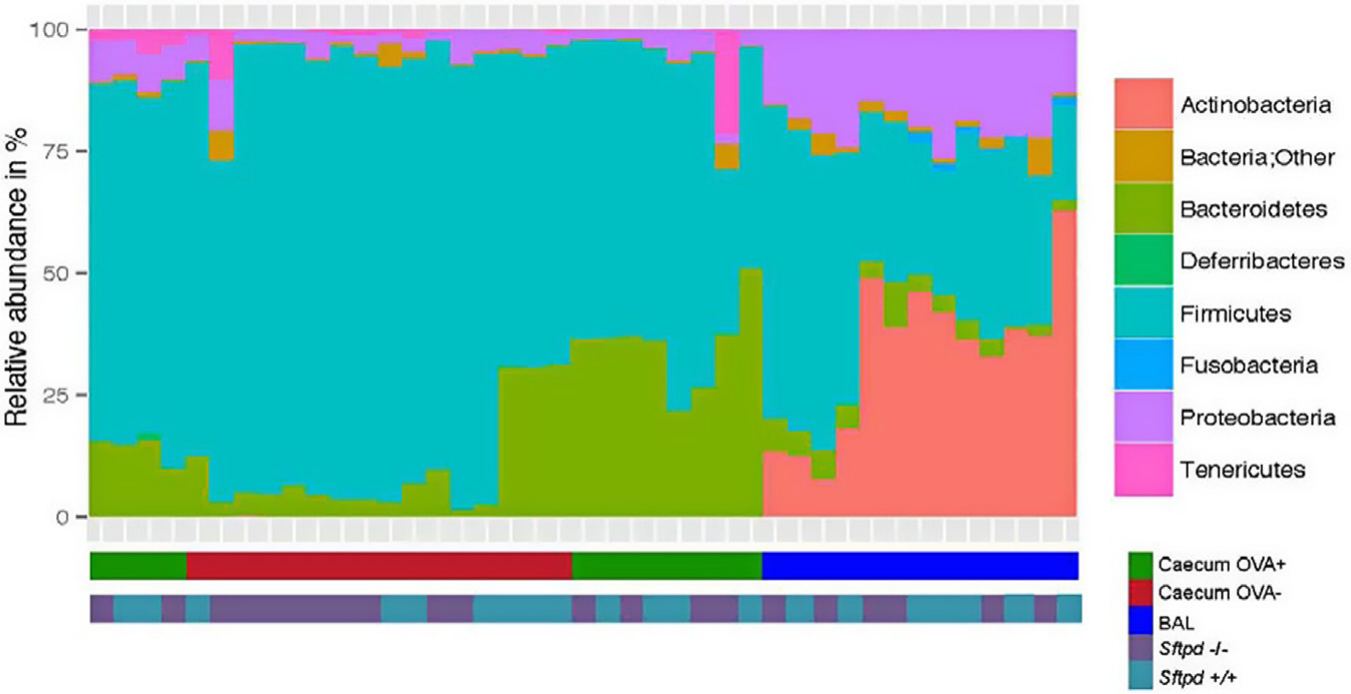
The overall phyla composition in caecal and BAL samples from individual C57BL/6 N female *Sftpd+/+* and *Sftpd-/-* mice. Fig. 1 shows the phyla composition on the Y-axis as relative abundance. The 34 samples are clustered along the X-axis according to similarity between samples. The BAL samples are BLUE and the caecal samples are GREEN (OVA treated animals n = 16) or RED (for unchallenged animals n = 18). At the bottom, sample genotype is noted either *Sftpd−/-,* or *Sftpd+/+,* which shows no clustering. The caecal samples contained primarily *Firmicutes*, *Bacteroidetes*, *Proteobacteria* and minor occurrence of *Tenericutes* as well as *Deferribacteres*. In the BAL the bacterial community were composed of *Actinobacteria*, *Firmicutes*, *Proteobacteria* and Bacteroidetes together with *Fusobacteria.*

After sequence retrieval and initial data treatment 869802 reads were divided into 697 Operational taxonomic units (OTUs) with a median sequence distribution of 13685 sequences per sample. The caecal samples contained primarily *Firmicutes*, *Bacteroidetes*, *Proteobacteria* and minor occurrence of *Tenericutes* as well as *Deferribacteres* (Fig. 1). In the broncho-alveolar lavage (BAL) fluids the bacterial community were composed of *Actinobacteria*, *Firmicutes*, *Proteobacteria* and *Bacteroidetes* together with *Fusobacteria* as previously observed in other mouse strains and studies [32, 48].

### 2.2 The microbial communities do not change with the SP-D deficiency but OVA sensitization and lung challenge alters caecum community composition

**Fig. 2:**
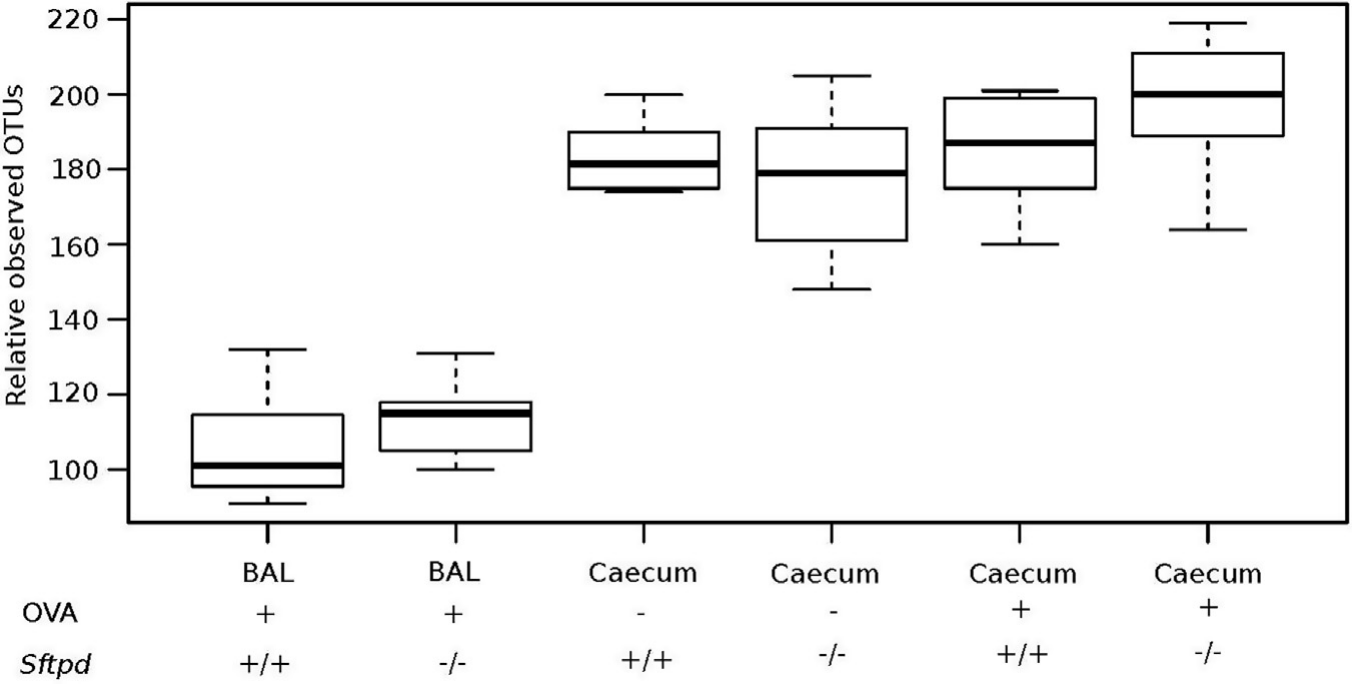
Bacterial richness. Relative observed OTUs in BAL and caecum from different experimental groups. The number of OTUs relative to each other from the different experimental groups at even sequencing depth with added variation bars. There is a significantly more OTUs in caecum samples regardless of mouse genotype and treatment compared to OTUs from BAL samples from OVA treated mice (Wilcoxon test, p < 0.001).

As demonstrated previously, OVA sensitization and challenge induced an allergic phenotype in the C57BL6 N mice with more pronounced mucous cell metaplasia in *Sftpd-/-* mice compared to *Sftpd+/+* littermates [21]. In order to elude microbial differences between microbiotas according to genotypes and airway disease treatments, we compared the number of OTUs found in the samples. Fig. 2 shows the number of observed OTUs from all our experimental groups and variation between samples (alpha diversity). The number of OTUs in caecal samples were 2 fold higher than OTU numbers found in the lungs (Wilcoxon test, p < 0.001). There were no differences in OTU levels between genotype or treatments in either the caecum or lung.

**Fig. 3:**
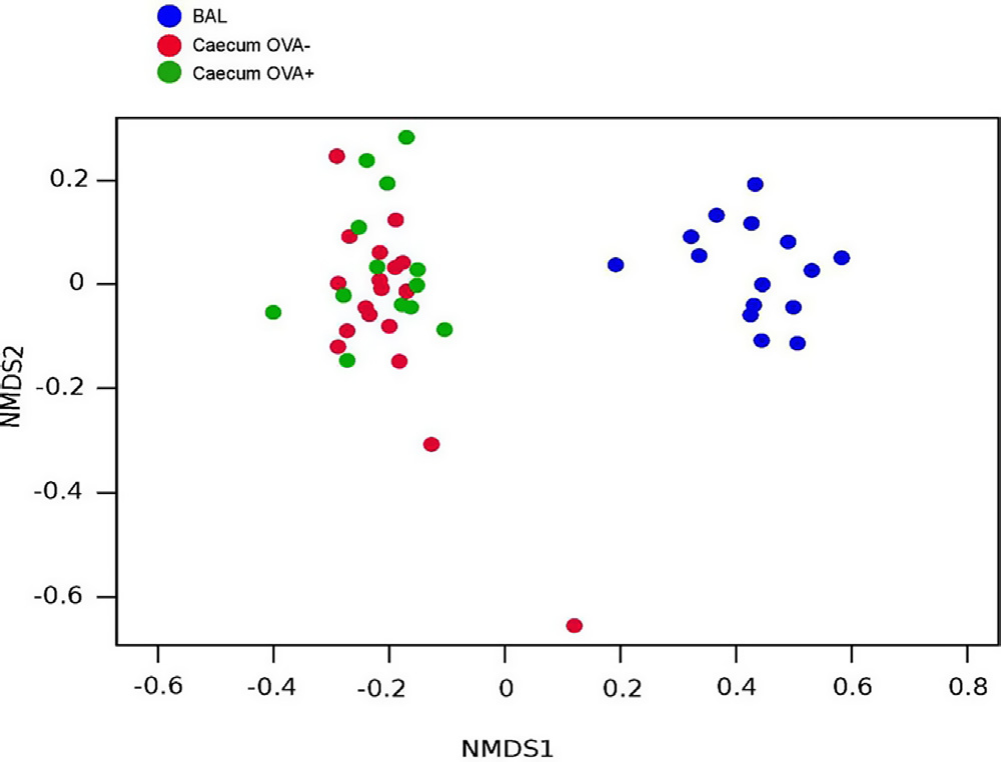
Microbial community clustering among experimental groups. Fig. 3 shows a non-metric multidimensional scaling (NMDS) plot of the beta diversity of all the individual samples regardless of genotype. There is a strong statistical significant dissimilarity between BAL (Blue n = 16) and caecum samples (Red n = 16 and Green n = 18) using the Anoism test (R = 0.898, p = 0.001). There is also a statistical significant difference between caecum samples from OVA treated animals (Green) and non-challenges animals (Red) (R = 0.100, p = 0.038).

**Fig. 4:**
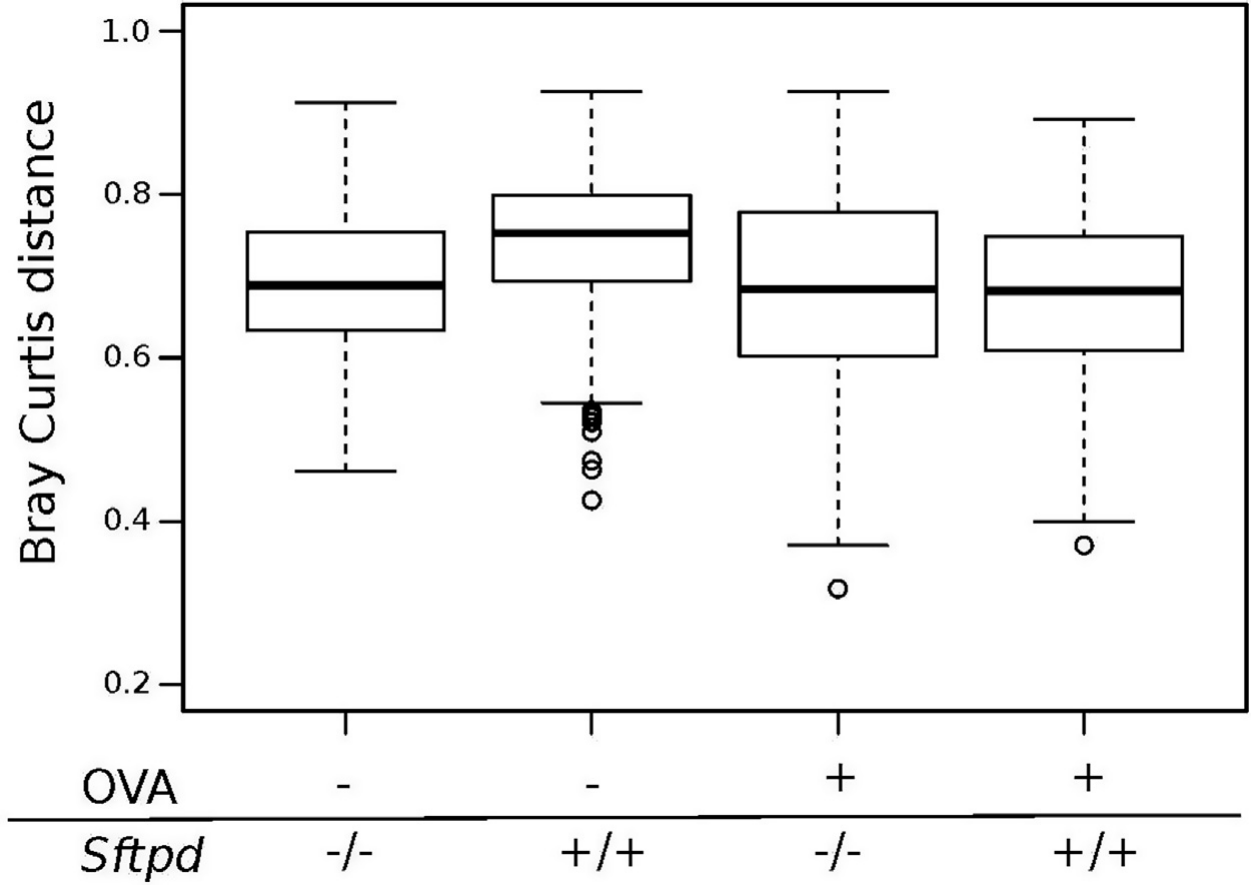
SP-D gut microbiota samples are more variable. Fig. 4 shows the Bray Curtis distance, which describes how different the caecum samples are within each experimental group compared to knockout naïve animals. (OVA- *Sftpd* −/-) and OVA treated groups (OVA+ *Sftpd−/-,* OVA *Sftpd+/+*), wild type (OVA- *Sftpd+/+*) animals without OVA exposure have the largest variation between mice (Kruskal-Wallis test, P-value = 1.32e-07).

We also investigated how different OTUs are distributed among all samples (beta diversity) (Fig. 3) and in caecal samples only. All the caecum samples cluster together completely separated from the lung and the SP-D deficient genotype did not discriminate between samples. The naive wildtype (*Sftpd+/+*) gut microbiota did have the statistical significant largest bacterial variation, compared to both naïve *Sftpd-/-* and allergic airway disease groups (Kruskal-Wallis test, P-value = 1.32e-07) (Fig. 4).

**Fig. 5:**
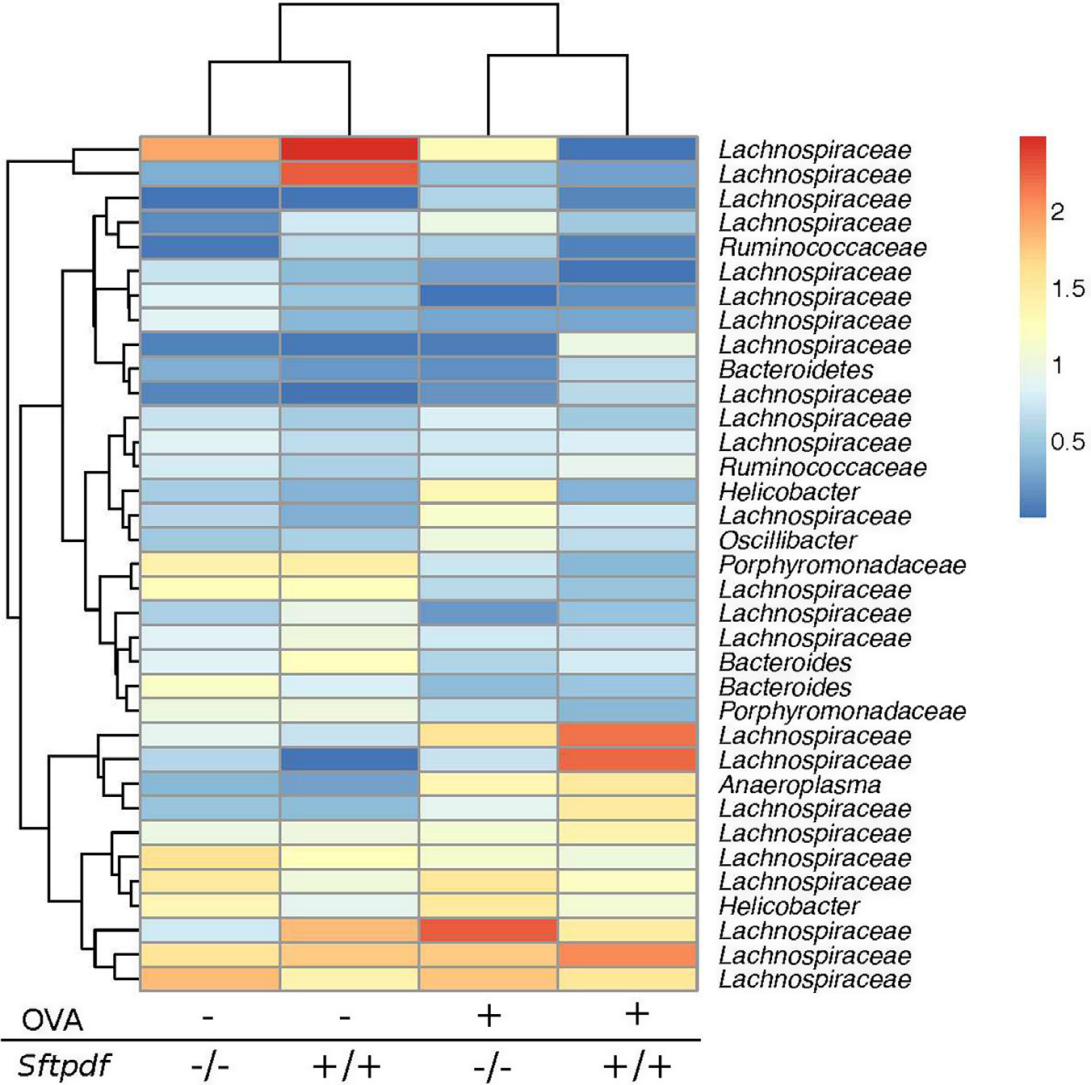
Primary observed bacterial species in the gut summarized by treatments. Fig. 5 shows a heatmap of the primary bacterial species (>5% relative frequency) summarized by treatment. The OVA treatments only are significantly different from naïve animal (R = 0.100, p = 0.038). The primary differences lies within the OTUs from bacterial family of *Lachnospiraceae.*; Shown are OTUs with an average frequency > 5%. Data was log-transformed. Non-challenged animals (OVA-) and OVA treated animals (OVA+).

The gut microbiotas from OVA treated mice cluster together (Fig. 3), slightly separate from the control animals, regardless of genotype in a statistical significant manner (Anoism R = 0.100, p = 0.038). So, we investigated which OTUs from the gut microbiota that discriminated between control and OVA sensitized and challenged mice. The heatmap in Fig. 5 shows the primary bacterial species in ceacum samples with an average frequency >5%. The primary differences lie within the OTUs from bacterial family of *Lachnospiraceae,* but also species within family *Ruminococcaceae* and the genus of *Helicobacteraceae* contribute.

### 2.3 The lung microbiota under OVA conditions

**Fig. 6:**
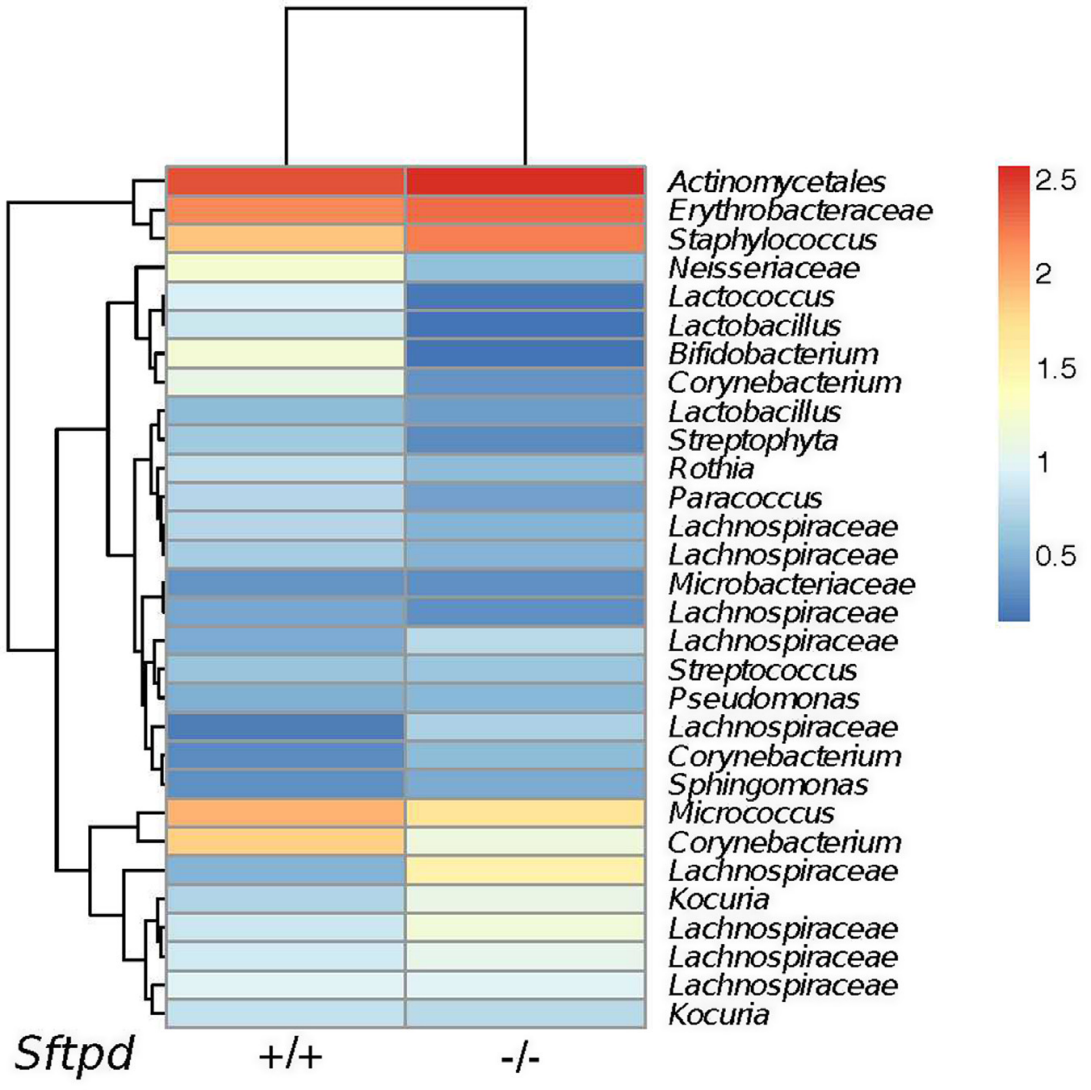
Lung microbiota summarized by genotype. Fig. 6 shows bacterial species distribution distinguished between *Sftpd* −/- and *Sftpd* +/+ from BAL samples. Most common bacterial OTUs observed in average above 5% (Data are log-transformed) There is no statistical significant clustering between genotypes samples based on the meta data in the BAL samples confirmed by the anosim test R = 0.03304, = 0.322.

**Fig. 7:**
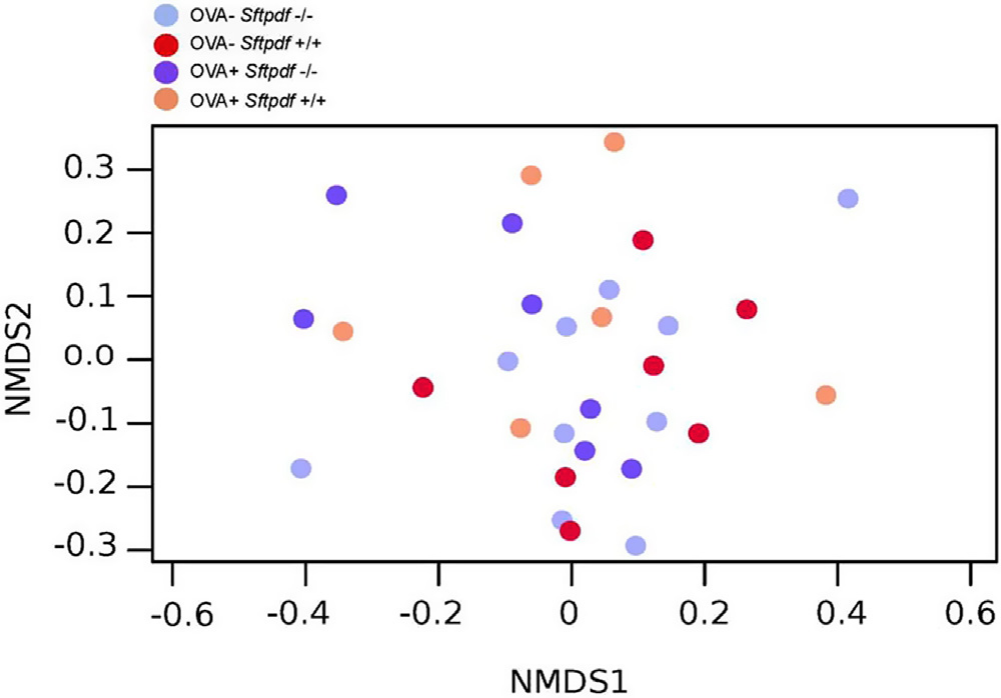
Cluster analysis of the beta variation between caecal samples. Fig. 7 shows beta variation between all caecum samples based on OVA treatment and genotype. There are no statistical significant differences of the bacterial communities between genotypes in either treatment group using anoism. (OVA- *Sftpd*−/- VS. OVA- *Sftpd*+/+, R = -0.018, P = 0.541) (OVA+ *Sftpd* +/+ VS. OVA + *Sftpd* −/-, R = −0.06614, P = 0.709).

**Fig. 8:**
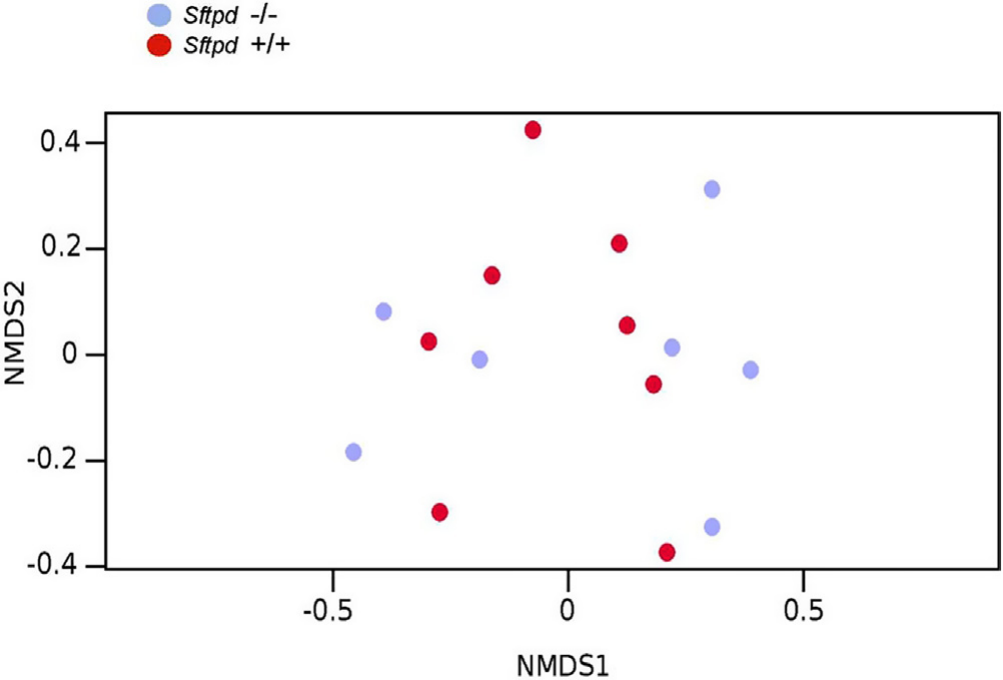
Cluster analysis of the beta variation between BAL samples. Fig. 8 shows beta variation between BAL samples according to genotype. There is no statistical significant clustering between the BAL samples based on the *Sftpd* −/- or *Sftpd +/+* using meta data in the BAL samples confirmed by the Anosim test R = 0.03304, = 0.322.

The lung microbiota (Fig. 6) is clearly separate from the gut as seen in Fig. 1, Fig. 2 and Fig. 3. But there was no clustering in beta diversity (Fig. 7) of the lung microbiota according to SP-D-genotype. This was confirmed by the Anosim test (R = 0.03304, = 0.322) (Fig. 8). We also tested how different the BAL samples are within each genotype. The comparison of similarities between knockout and wild type samples, shows that there is no statistical difference between groups (Wilcoxon test, p > 0.05)

**Fig. 9:**
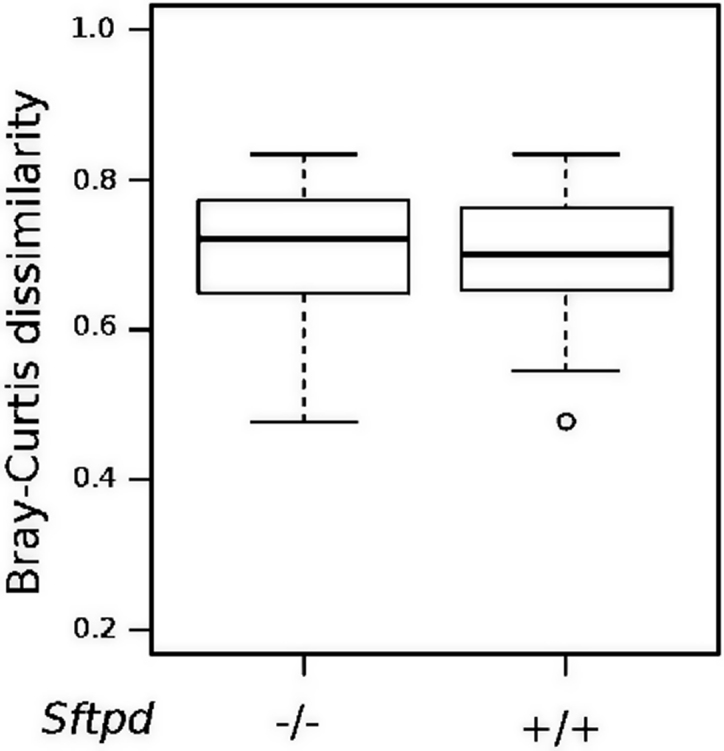
Bray-Curtis dissimilarity between BAL samples according to genotype. Fig. 9 shows how different the BAL samples are within each genotype. The comparison of similarities within the BAL groups samples (*Sftpd −/- =* Knockout, *Sftpd +/+* = wild type) shows that there is no difference (Wilcoxon test, p > 0.05).

There was no difference in similarities within genotypes (Wilcoxon test, p > 0.05) (Fig. 9).

Finally, we compared the most common bacterial OTUs observed in the lung microbiotas (Fig. 6). Even though beta diversity does not discriminate between the SP-D genotypes in the lung microbiota there are still some differences in OTUs primarily at family level. The heatmap reveals differences in several biological relevant phylogroups such as *Staphylococcus, Lactobacillus and Bifidobacterium.*

## 3 Discussion

Our study presents the first description of the murine microbiome in SP-D deficient model with induced allergic airway disease using NGS 16S rRNA gene sequencing. Based on known differences in the innate immune system of the *Sftpd-/-* mouse, our original hypothesis was that lack of SP-D protein could infer alterations in microbiota able to overcome the co-housing effect. In the SP-D-deficient model the mice are bred from heterozygous parents all genotypes mixed and co-housed. A significant community shift in gut microbiota was observed after challenge with OVA compared to naïve mice, but the microbial communities of the cohoused mice were not significantly different according to mouse genotype. The answer to this question is very important since any genotype specific microbiota changes in themselves could have an impact on the outcome of inflammatory disease experiments in the SP-D model and influence the analysis. We have previously shown that OVA treatment in itself has a clear impact on the lung microbiota in wildtype BALB/cJ mice [47].

Our results on gut microbiota show that the naïve wild type has the largest variation in OTUs within the experimental groups. This could indicate that OVA-sensitization and challenge that induce inflammation also reduces variation in microbiota composition. It is worth noting that there were only mild allergic differences between OVA-sensitized and challenged *Sftpd+/+* and **S*ftpd−/-,* [21] making it possible to separate the effects of SP-D and allergy on the gut microbiota.

The lung microbiotas of OVA sensitized and challenged animals cluster completely separate from the gut, with significantly lower numbers of OTUs per sample. There was no difference in similarities between genotypes in the OVA sensitized and challenged animals. This aligns with previous observations from analysing the unchallenged lung microbial community of *Sftpd-/-* and *Sftpd+/+* mice with the faster but less sensitive DGGE method [21]. A strength of this study would have been to have compare BAL samples from both sexes of naïve animals to those of OVA challenged mice with 16S rRNA gene sequencing. We have previously shown that DGGE is suitable to analyse microbial shifts and sex dependency in lung microbiota [49]. Sex difference in lung microbiota, under OVA allergy and control conditions, is thus an example of a difference capable of overcoming co-housing effects [47].

The primary intestinal bacterial differences in our experiments between OVA and unchallenged mice are found within the OTUs from bacterial family of *Lachnospiraceae*, which are commonly found in the GI-tract of mammals including humans, where they participate in the production of SCFAs [50]. A change in SCFA producing species in the gut can influence local allergic inflammation in the lung, possibly in a bi-directional manner [51]. The gut-lung crosstalk is mediated through primed regulatory T-cells (T-regs) [52, 53]. Such observation were recently supported by studies demonstrating that gut microbiota modulated by the presence of intestinal helminths, increases in SCFA producing species and SCFA production and that transfer of the modulated gut microbiota in itself can mediate protection against induced allergic asthma in mice [46]. The opposite direction of effects is less well explored. However, recent results obtained using airway LPS administration or the house dust mite (HDM) model of allergic airway disease in mice showed that the resulting lung inflammation changed the bacterial composition of the gut [40, 54]. Our results are in support of those previous data and suggest that pulmonary inflammation can alter the composition of the gut microbiome through yet unidentified pathways.

## 4 Conclusions

The SP-D deficient genotype does not cause alterations to the microbiota that interfere with the use of the SP-D deficient mice for immunological research. The genotype does not significantly alter intestinal microbiotas in control conditions or either lung or gut microbiotas in OVA induced allergic airway disease. However, OVA sensitization and pulmonary challenge, does alter the composition of gut microbiota, supporting a previously reported bidirectional lung-gut crosstalk in a HDM allergy model. The data supports that lung, and cecal microbiotas are very dynamic that the gut-lung microbial axis is bi-directional. Investigations of cross talk and mechanistic effects of induced allergic airway disease on intestinal microbiota are warranted.

## 5 Methods

### 5.1 Mouse model

Six- to 8-weeks old C57BL/6 N female *Sftpd+/+* and *Sftpd-/-* [14] littermate mice were bred from *Sftpd+/−* heterozygous parents [21]. They were co-housed in the animal house at the University of Southern Denmark with access to pelleted food and water ad libitum. Test for zygosity was performed on tail biopsies of 3-week-old mice using the REDExtract-N-AmpTM Tissue PCR Kit (Sigma-Aldrich) according to the manufacturer’s instructions. SP-D genotypes were identified by multiplex PCR using the 5′-GGTTTCTGAGATGGGAGTCGTG-3′ as the forward primer, and 5′TGGGGCAGTGGATGGAGTGTGC-3′ and 5′GTGGATGTGGAATGTGCGAG-3′ reverse recognizing the wild-type allele and the *Sftpd*-deficient alleles, respectively.

All animal experiments are in accordance with Council of Europe Convention European Treaty series 123 and the Danish Animal Experimentation Act (LBK 1306 of 11.21.2007). All protocols and procedures were approved by the Danish Animal Experiments Inspectorate procedures (ref. no. 2012-15-2934-00525)

### 5.2 OVA induced allergic airway disease

*Sftpd+/+* and *Sftpd-/-* mice were randomized into two experimental groups: OVA (n = 16) and control groups (n = 18). Mice were sensitized on day 0 and day 7, received 50 μl PBS intranasally on day 12 and day 13, challenged on days 14–16 and sacrificed on day 17. For sensitization of the OVA group, 20 μg OVA precipitated with 2 mg alum in 200 μl PBS was administered by intraperitoneal injection. Mice were then challenged by intranasal administration of 20 μg OVA in 50 μl PBS under light isoflurane anesthesia [21]. The control group was sensitized with alum in PBS and challenged with PBS instead of OVA.

### 5.3 Bronchoalveolar lavage (BAL)

BAL was performed with 0.5 ml of sterile PBS and gently aspirating back and forth after 30 s (4 times). After centrifugation at 825 g for 10 min at 4 °C, the cell free supernatant was stored at −80 °C for bacterial analysis. If recovered BAL was less than 75% of the original PBS, it was excluded.

### 5.4 Sampling and DNA extraction

Caecum samples were taken from the animals last to avoid cross contamination. The caecum was cut open and approximately 50 mg stool was removed using sterile plastic loops directly into cryo tubes and snap frozen in liquid nitrogen. DNA extractions from frozen caecal samples was done using Qiagen spin protocol for detection of pathogens from stool (Qiagen, DNA mini stool kit Denmark) and frozen cell free BAL samples were done using Qiagen spin protocol (Qiagen, DNA mini kit Denmark) as previously described [48].

### 5.5 Microbiome analysis

DNA extract (5 ng) was used to generate a 466 bp long amplicon fragment using the prokaryotic universal primer of 341F (5′-CCTAYGGGRBGCASCAG-3′) and the 806R (5′-GGACTACNNGGGTATCTAAT-3′). The PCR reaction mix (25 μl) contained 1 μl (12.5 μM) of each primer, 5 μl (5x) of the Phusion HF Buffer (Finnzymes, Vantaa, Finland), 0.5 μl (10 mM) of dNTPs, 0.25 μl of the (0.5 Units) Phusion DNA polymerase (Finnzymes, Vantaa, Finland) together with 1 μl template and 16.25 μl sterile Sigma water. Target fragments were amplified using the following conditions: 98 °C for 30 s, followed by 30 cycles of 98 °C for 5 s, 56 °C for 30 s and 60 °C for 1 min and a final extension of 72 °C for 5 min. In a second PCR round, sequencing adaptors and barcodes were attached to the amplicons under the same reaction condition as above with a lowered cycle number of 15. Furthermore the PCR products were purified and cleaned using Agencourt AMPure XP beads. After pooling the normalized amplicon libraries, sequences were generated with the MiSeq 2 × 250 Nextera KIT v2 cartridge (Illumina).

The generated sequences were first de-multiplexed and paired followed by a primer truncation and low quality removal step using the default setting of the Uparse pipeline [55]. Chimeric sequences were discovered with Uchime and disregarded [56]. Afterwards, OTUs were picked with Usearch at 97% sequence identity [57] and classified using Mothur (v.1.33.3) and the RDP database [58]. To deal with variation in sequences depth the OTU proportion were corrected using the zero-inflated Gaussian distribution implemented in MetagenomeSeq [59].

### 5.6 Statistical analyses

Data were compared using the non-parametric Wilcoxon Rank-Sum test (when comparing two sample or matched samples) or the Kruskal-Wallis test. Differences were considered significant with a *p*-value < 0.05. Treatment effects on the overall microbial community structure was evaluated by generating the Bray-Curtis dissimilarity between samples and clustering was visualized by using ordination applying non-metric multidimensional scaling (NMDS) generated in the R vegan package [60].

The microbial clustering was further evaluated with the analysis of similarity (Anosim) [61] and tested for significance by 999 permutations with a 5% significance level. Individual variation of selected microbes was displayed with the Euclidean distance in the heatmap based on the log-transformed metagenomeSeq normalized OTU counts.

## Declarations

### Author contribution statement

Kenneth K. Barfod: Conceived and designed the experiments; Analyzed and interpreted the data; Contributed reagents, materials, analysis tools or data; Wrote the paper.

Michael Roggenbuck: Conceived and designed the experiments; Performed the experiments; Analyzed and interpreted the data; Wrote the paper.

Suzan Al-Shuweli, Dalia Fakih: Performed the experiments.

Søren J. Sørensen: Conceived and designed the experiments; Performed the experiments.

Grith L. Sørensen: Conceived and designed the experiments; Analyzed and interpreted the data; Wrote the paper.

### Funding statement

This work was supported by Innovation Fund Denmark; Statens Serum Institut; National Research Centre for the Working Environment and DuPont.

### Competing interest statement

The authors declare no conflict of interest.

### Additional information

No additional information is available for this paper.
